# Neural self-organization during episodic encoding: deep recurrent effective connectivity from source-localized EEG

**DOI:** 10.3389/fpsyg.2026.1766795

**Published:** 2026-03-23

**Authors:** Hrishikesh Pable, Martin D. Pham, Maryam Mehri Dehnavi, Robin Chhabra, Amedeo D’Angiulli

**Affiliations:** 1 Neuroscience of Imagination, Cognition and Emotion Research (NICER) Lab, Carleton University, Ottawa, ON, Canada; 2 ParaMathics Lab, Department of Computer Science, University of Toronto, Toronto, ON, Canada; 3 ELIXIR Lab, Mechanical, Industrial, and Mechatronics Engineering Department, Toronto Metropolitan University, Toronto, ON, Canada; 4 Department of Neuroscience, Carleton University, Ottawa, ON, Canada

**Keywords:** Default Mode Network (DMN), effective connectivity, episodic encoding, Low-Resolution Brain Electromagnetic Tomography (sLORETA), mental visual imagery, recurrent neural network, Salience Network (SN), source-localized electroencephalography (EEG)

## Abstract

Understanding dynamic, directional interactions among large-scale brain networks which support sensory-based cognition remains a major challenge. Focusing on neural dynamics during perceptual encoding in a supraspan immediate free-recall paradigm, we develop a computational framework for estimating effective connectivity from source-localized electroencephalography (EEG). We integrate standardized Low-Resolution Brain Electromagnetic Tomography (sLORETA) with a biologically-informed recurrent neural network. The model incorporates key neurobiological constraints including Dale’s Law, separate excitatory and inhibitory populations, and population-specific time constants to provide greater interpretability than conventional black-box machine learning approaches. We apply this framework to the study of the large-scale triple-network framework – Salience Network (SN), Default Mode Network (DMN), and Task-Positive Network (TPN) – comparing bidirectional interactions during rest and the encoding phase of a free-recall task of two distinct stimulus types (geometric shapes and words). The inferred connectivity patterns reveal state-dependent and bidirectional influences, in particular, SN-driven facilitation of both DMN and TPN during recall, stronger TPN–DMN inhibition during rest, and a reversal in DMN-to-SN influence across states. We supplement the computational findings by uncovering the mediating influence of individual differences in mental visual imagery as measured by the Vividness of Visual Imagery Questionnaire (VVIQ). Specifically, we uncover two selective latent associative links between VVIQ and the connectivity strength from SN directly and indirectly (after DMN modulation) to TPN, for geometric shapes and word encoding, respectively. The inferred connectivity patterns challenge switching-based accounts and highlight deep recurrent effective connectivity as a novel way to gain insights into the self-organization dynamics involved in episodic encoding.

## 1 Introduction

### 1.1 Motivation

Electroencephalography (EEG) provides a non-invasive means of tracking the brain’s rapid neural dynamics, offering millisecond temporal resolution essential for studying cognitive processes such as perceptual encoding during memory formation (Squire and Wixted, 2011). Yet interpreting EEG at the systems level requires moving beyond sensor-space activity to the coordinated behavior of large-scale networks. Source localization methods such as standardized Low-Resolution Brain Electromagnetic Tomography (sLORETA) (Pascual-Marqui, 2002) help overcome spatial resolution limitations of scalp EEG by estimating cortical generators, enabling investigation of network-level interactions with greater anatomical specificity. These advances are especially relevant for contemporary accounts of cognition that view the brain as a self-organizing system, in which higher-order functions arise from dynamic reconfigurations among distributed networks rather than isolated regional activations (Gerstner et al., 2014).

### 1.2 Background

A central framework in this literature is the triple-network model (Menon, 2011; Sridharan et al., 2008). The Default Mode Network (DMN) supports internally oriented thought, including autobiographical memory (Raichle et al., 2001), while the Task-Positive/Central Executive Network (TPN/CEN) is engaged during externally directed, attention-demanding behavior (Demertzi and Whitfield-Gabrieli, 2016). The Salience Network (SN) is proposed to regulate transitions between these systems by detecting behaviorally relevant events and reallocating processing resources (Sridharan et al., 2008). Although fMRI research has revealed reliable patterns of DMN–TPN antagonism, the causal mechanisms through which these networks self-organize during cognitive engagement remain unclear (Lefebvre and D’Angiulli, 2019). Standard functional connectivity methods capture statistical associations but do not specify how one network exerts directional influence over another, limiting insight into the competitive and cooperative dynamics that shape cognition. To move beyond descriptive associations, it is critical to quantify effective connectivity: the directed influence that one neural system exerts over another (Friston et al., 2003). As a possible solution, contemporary machine learning approaches, including recurrent neural networks (RNN), can in principle capture complex temporal dependencies. However, they typically function as opaque black boxes, optimizing predictive performance without providing mechanistic or physiologically interpretable accounts of how large-scale networks interact (Grossberg, 2020).

Recent intracranial EEG work has begun to characterize triple-network dynamics during human episodic memory with a level of temporal and causal precision that complements our source-localized EEG approach. Using depth recordings from 177 participants across four verbal and spatial memory tasks, Das et al. showed that the anterior insula, a key salience network node, exerts robust directed influence on both default mode and frontoparietal networks during both encoding and recall, with task-specific suppression of high-gamma activity in posterior cingulate/precuneus and strong replication across experiments (Das et al., 2024). At rest, complementary multicohort iEEG analyses have demonstrated that the salience network exhibits stronger intra-network coupling, faster decorrelation, higher entropy, and greater net causal outflow than either default mode or frontoparietal networks, supporting its role as a flexible switching hub (Das and Menon, 2020). Focusing on the default mode network, intracranial depth recordings have further revealed that slow-wave intra-DMN synchrony and beta-band cross-network interactions reconfigure between resting state, memory encoding, and recall, indicating that DMN effective connectivity itself is dynamically modulated by mnemonic demands (Das et al., 2022). More locally within the salience system, recent work in humans performing a spatial episodic memory task has shown that insular traveling waves propagate in distinct spatial patterns across task phases and that these waves modulate interactions among insular subregions and predict trial-by-trial memory success, linking mesoscopic insular dynamics directly to episodic performance (Das et al., 2025). Converging evidence from intracranial recordings during reward learning shows that the human insula also leads dorsomedial prefrontal cortex in communicating both signed and unsigned reward prediction errors, underscoring its broader role as a causal outflow hub in cognitive control circuits (Hoy et al., 2023). Finally, translational work in mouse models illustrates how molecular perturbations in components of auditory circuitry (e.g., Embigin–Cdh23 interactions) can produce distributed brain and behavioral phenotypes (Newton et al., 2023), highlighting the importance of mechanistic frameworks that link cellular and network-level dynamics; together, these studies motivate the biologically constrained neural-mass modeling and triple-network effective connectivity framework developed here.

### 1.3 Terminology

To ensure conceptual clarity, we operationalize the following key terms as used throughout this manuscript:

1. *Self-organization* refers to the data-driven emergence of coordinated network patterns without externally imposed interaction rules (Sugimoto et al., 2025). In our framework, network coupling strengths are learned from temporal dynamics rather than specified *a priori*, allowing interaction patterns to organize based on the statistical structure of neural activity.
2. *Emergence* describes network-level properties that arise from—but are not explicitly encoded in—the regional dynamics (Miller et al., 2024), which relies on identifying (Wein et al., 2021) independent functional networks. It has been shown that there is reduced emergent neural dynamics in patients with disrupted connectomes (Luppi et al., 2023). Our network couplings are determined by the triple network model networks (and a fourth network for all other regions) trained on the time series data of parcellated regions, capturing large-scale coordination patterns not directly observable at the parcel level.
3. *Mechanistic* indicates that our model embeds known neurobiological principles such as Dale’s Law (Harris et al., 2023), population-specific time constants and excitatory-inhibitory balance into the architecture. Unlike black-box models, the learned parameters have direct physiological interpretation as synaptic coupling strengths and temporal response properties.
4. *Causal/directional influence* refers to predictive temporal dependencies rather than interventional causality (Wismüller et al., 2021). The learned connectivity between functional networks captures how activity in one network at time *t* predicts changes in another network at *t + 1*, constrained by neural mass dynamics. This represents model-based effective connectivity: directional influences that optimize temporal prediction within biological constraints, distinct from causal claims requiring experimental manipulation. See (Friston et al., 2003) for comparison with Dynamic Causal Modeling (DCM).

### 1.4 Contributions

We develop a biologically informed neural-mass modeling framework to infer effective connectivity among large-scale brain networks directly from source-localized EEG during episodic memory encoding. Neural-mass models describe the collective dynamics of interacting excitatory and inhibitory neuronal populations (Wilson and Cowan, 1972) and allow inference of directed coupling parameters that reflect mechanistic interactions (Paszke et al., 2019). By embedding the biophysical principles within a recurrent architecture, the model learns connectivity patterns that best explain the observed temporal evolution of neural activity. The resulting connectivity estimates therefore reflect model-based effective connectivity, i.e., directional influences that optimize the prediction of network dynamics under biologically constrained neural-mass interactions.

Our framework combines theory-driven network definitions with data-driven inference of interactions among them. The anatomical boundaries of the large-scale networks are specified *a priori* based on established neuroanatomical atlases and cognitive network frameworks (Menon, 2011; Glasser et al., 2016). In contrast, the directional interactions between these networks are inferred directly from the data without imposing assumptions about their connectivity structure. This hybrid strategy preserves the interpretability of established brain network organization while allowing flexible identification of the dynamical coupling patterns that govern their coordinated activity.

Especially, this approach differs conceptually from established causal modeling frameworks such as (DCM) (Friston et al., 2003) that relies on explicit mechanistic hypotheses about network architecture and uses Bayesian inference to compare predefined models. In contrast, our method allows directional dependencies to emerge from the data while constraining the model with biologically plausible neural dynamics. The inferred connectivity matrices therefore capture optimized directional interactions that explain observed temporal activity patterns, rather than formal causal claims about the underlying neural circuitry.

We apply this approach to investigate large-scale network dynamics during the encoding phase of a memory task. We use a supraspan immediate free-recall paradigm in which participants encode lists of stimuli (words and geometric shapes) that exceed short-term memory capacity and then attempt free recall. While the behavioral recall measure reflects contributions from both working memory and long-term episodic processes, our EEG analyses focus exclusively on the encoding period. According to current mainstream theory in cognitive psychology, the episodic buffer supports the integration of items and contextual information (Baddeley et al., 2021; Hitch et al., 2025). Encoding supraspan material partly engages episodic encoding mechanisms such as attention, inter-item association, and context binding in working memory (Baddeley et al., 2019). Therefore, this paradigm provides an appropriate framework for studying the neural network dynamics of episodic encoding (or episodic buffer processes).

Finally, we examine whether the inferred connectivity patterns relate to individual differences in visual imagery ability, a cognitive factor increasingly recognized for its influence on episodic encoding and retrieval (Molokopoy and D’Angiulli, 2022). This integrative approach links fine-grained temporal dynamics, large-scale network organization, and psychological variability, providing a mechanistic account of how distributed brain networks reorganize during episodic memory.

### 1.5 Paper organization

The remainder of this paper is organized as follows. Section 2 describes the experimental paradigm, EEG data acquisition protocol, and our computational framework, including source localization procedures, the biologically-informed neural mass model architecture, and statistical methods for connectivity inference. Section 3 presents the main results, including behavioral performance, inferred network connectivity patterns across task and rest conditions, baseline model validation, and exploratory correlations with individual differences in visual imagery ability. Section 4 discusses the theoretical implications of our findings for the triple-network model, compares our approach with conventional methods, and acknowledges methodological considerations. Supplementary materials provide additional technical details and validation results.

## 2 Materials and methods

### 2.1 Experiment

The foundation of our study is a supraspan immediate free-recall experiment on human adults (Kahana, 1996) with concurrent EEG recording during the encoding phase. Participants studied lists of words and geometric shapes that substantially exceed typical working-memory capacity (16 items per condition) and then immediately attempted to freely recall as many items as possible. Although immediate free recall without a distractor period involves both working-memory and episodic retrieval processes at the behavioral level, the encoding of supraspan lists can be assumed to partly engage episodic encoding mechanisms including inter-item association formation and item-context binding (Howard and Kahana, 2002; Baddeley et al., 2019, 2021; Hitch et al., 2025). Critically, our EEG analyses focused exclusively on the encoding/presentation phase, not the retrieval phase. The experiment consisted of several blocks, alternating encoding trials with free recall tasks and resting-state periods, presented in a counterbalanced order across participants and following a within-subjects design.

#### 2.1.1 Participants

A cohort of 15 healthy, right-handed adults (mean age = 20.13 years, SD = 1.92; 11 females, 4 males) was recruited from Carleton University via the SONA participant pool system and provided written informed consent. Upon completing an intake demographic questionnaire and a brief questionnaire about past drug use and medical history, participants reported no history of neurological or psychiatric disorders, no motor, coordination or language disability or disorder, and normal or corrected-to-normal vision. Participants averaged 7.13 h of sleep per night (SD = 1.22), with 2 participants reporting sleep issues. One participant reported past cannabis use, and one reported current cannabis use; all other participants reported no drug use in the past three months. Excluding these participants had no effect on overall group statistics, hence we included them in the final analysis.

Before starting the experiment, the participants also completed the Vividness of Visual Imagery Questionnaire (VVIQ) (Marks, 1973) to assess individual differences in mental imagery ability. The VVIQ consists of 16 items rated on a 5-point scale (1 = no image at all, 5 = perfectly clear and vivid as real seeing).

The study protocol was approved by the Carleton University Research Ethics Board (CUREB-B Clearance #111569). All participants provided written informed consent prior to participation and received 2% course credit compensation for their time. All data was anonymized.

#### 2.1.2 Procedure: free recall task and EEG data acquisition

**Figure 1:**
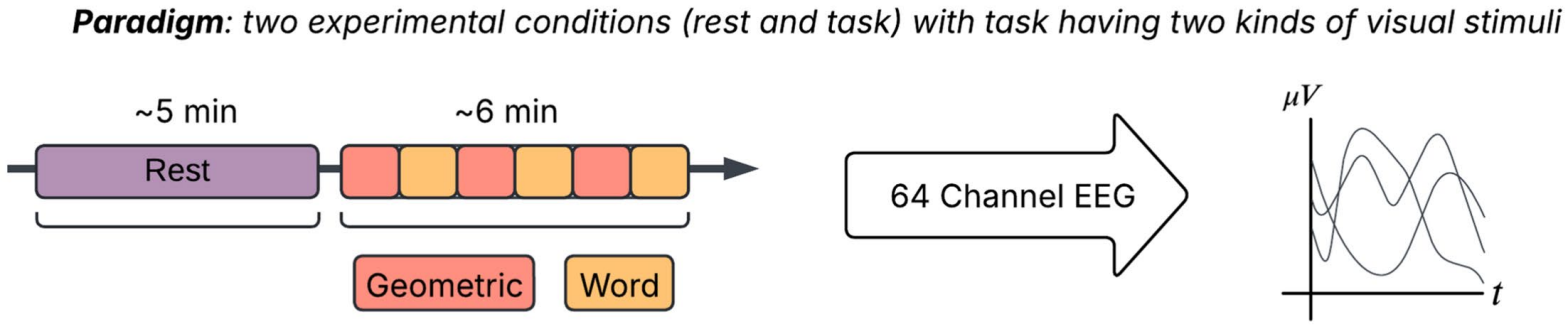
The participants are shown geometric shapes and words alternatively, and asked to remember them. The EEG signals are captured during this episodic encoding phase. Resting state data is the 5 min window in the above figure, and entire task (or simply task) refers to the subsequent 6 min window of visual stimuli (Geometric + Word).

Participants were seated in front of a computer screen where the experimental stimuli were presented (see Figure 1). A 64 channel acquisition system with recessed Ag–AgCl electrodes (10 mm each) mounted in an electrode cap (Quik-Cap, Neuroscan, Charlotte, NC, USA) amplified and referenced to a separate electrode located on the nose tip with AFz ground. The EEG montage was based on external landmarks of the skull (i.e., the inion, the nasion, the left and right preauricular points) and standard percentage distances as per 10–20 system. The Neuroscan EEG quick cap was fitted over the participant’s head, followed by calibration for individual electrical impedance levels. Also a routine pre-recording online inspection of resting EEGs (i.e., pre and post-experiment 2-min open/closed eyes) showed ordinary spectral frequency distribution typical of adults in all participants. After the cap was set up, a resting state measurement was recorded (5 min). During this time, participants were asked to remain still and to avoid thinking about anything in particular. Participants were then instructed that following repeated presentation of a variety of stimuli (both verbal and pictorial) on the screen, they had to freely recall as many of the stimuli as possible (See Supplementary Figures 1, 2).

Once participants declared they understood the task, they underwent a standard free recall experimental procedure (Aka et al., 2021) made up of a block composed of two stimulus conditions: a word list (16 items), and a list of geometric shapes (16 items). The task was divided into three repetition blocks, with each word or shape presented three times across blocks (see Figure 1). Each word was displayed for 2 s with a 1 s rest period between presentations. Stimulus presentation timing was matched across stimuli. Following each complete run of stimuli (96 total presentations across both conditions), participants freely recalled as many items as possible on a blank piece of paper using a pencil.

EEG data was recorded only during the resting-state periods and stimulus presentation (encoding) periods, not during the free recall periods. Our analyses therefore focus on neural network dynamics as participants encode information into memory, not on the retrieval processes themselves. Although the subsequent behavioral recall measure reflects both working-memory and episodic retrieval processes, the encoding of 96 stimuli presented in supraspan lists (far exceeding the ~4–7 item capacity of short-term memory) requires episodic encoding mechanisms such as formation of inter-item associations, binding to temporal and spatial context, and engagement of long-term memory consolidation processes (Howard and Kahana, 2002; Ward and Beaman, 2025). Our neural measures therefore capture the brain network dynamics supporting episodic encoding, even though the behavioral readout reflects mixed working memory and long-term episodic memory components.

The EEG acquisition protocol followed all recommendations on current EEG standards (Dash et al., 2017). EEG signals were amplified (gain of 10; range of ±200 μV, or 400 μV peak-to-peak; Accuracy 29.80 nV/LSB) and low pass filtered at 500 Hz via SynAmps RT with a sampling rate of 1,000 Hz. Acquisition filters were single-pole Butterworth, 6 dB per octave, 3 dB down at 500 Hz. All electrodes were referenced to a separate reference electrode, and all data were re-referenced to a common average reference node *Cz* (i.e., the average was subtracted from each electrode for each time point). For post-data acquisition, EEGs were averaged separately for each condition for each electrode. Timestamp markers for the start and end of each experimental block were recorded and used for subsequent data segmentation and quality validation. Electrode impedances were kept below 5 kΩ. An online bandpass filter of 0.1–100 Hz was applied during acquisition.

Trials contaminated by excessive peak-to-peak deflection (i.e., >100 or < −100 μV) at non-ocular electrode sites were excluded from the average. The proportion of rejected trials was less than 10% after artifact correction and removal. The post-data acquisition average signal from the electrodes was amplified and digitized with filter settings at 0.15 Hz (high pass) and 100 Hz (low pass).

### 2.2 Approach to EEG analysis

#### 2.2.1 EEG preprocessing and source localization

**Figure 2:**
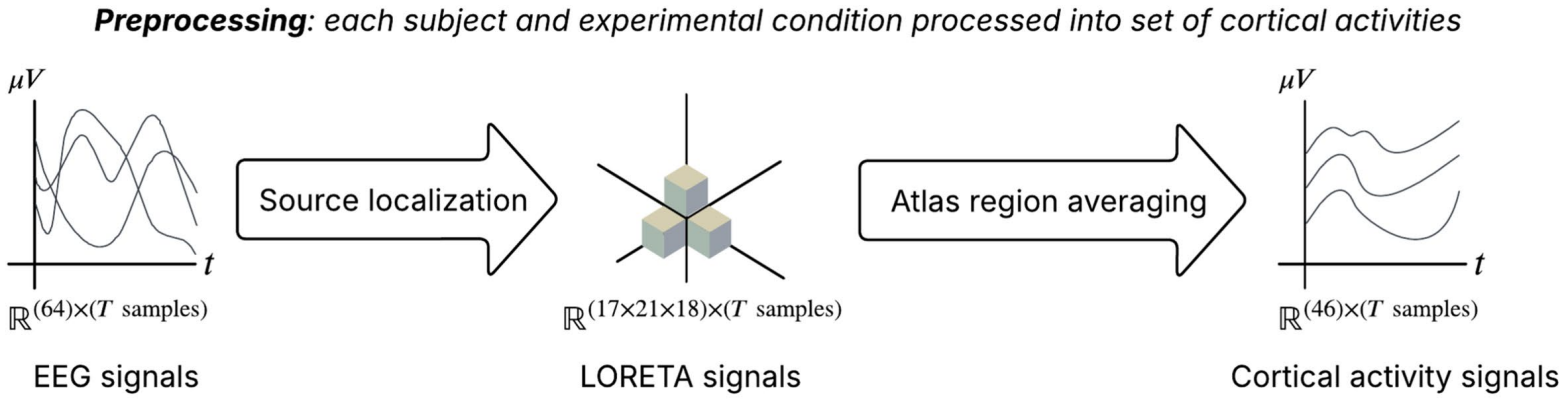
The preprocessing step transforms a set of EEG channels into a set of brain atlas based region channels of average activity.

Raw EEG data was processed using MNE-Python (Gramfort et al., 2013, 2014), including channel remapping to the standard Neuroscan1005 montage, removing non-EEG auxiliary channels (bipolar and horizontal eye movement channels), and application of average reference (Figure 2).

All subsequent source localization and connectivity analyses were performed on this broadband filtered data (0.15–100 Hz). We did not decompose the signal into specific frequency bands, as the neural mass model’s population-specific time constants naturally capture dynamics across multiple frequency ranges, and our focus was on characterizing overall directed effective connectivity rather than frequency-specific coupling mechanisms.

To estimate cortical source activity corresponding to EEG, we employed sLORETA (Pascual-Marqui, 2002). Typically, the “fsaverage” anatomical fMRI template is used to construct a three-layer boundary element model comprising brain, skull, and scalp compartments. A volumetric source space with 10 mm spacing is generated, and the forward model is computed using a minimum 5 mm constraint between each dipole source and the inner-skull surface to ensure numerical stability and physiologically plausible lead-field estimates.

**Figure 3:**
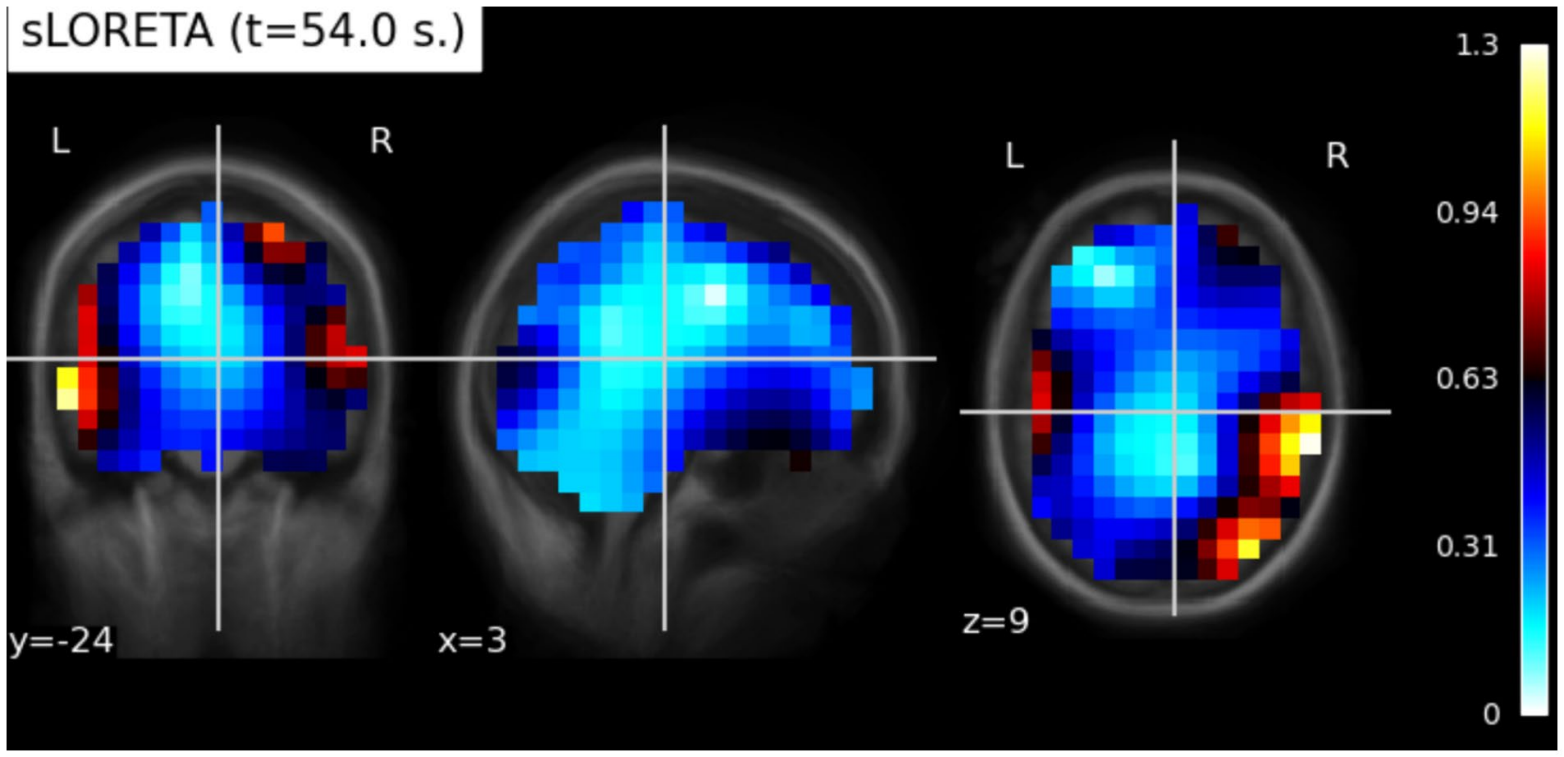
Source localization of EEG signals using sLORETA for subject 2 during encoding task at 54 s (generated using MNE Python library).

A noise covariance matrix was estimated directly from the raw EEG recordings to characterize the sensor-level noise statistics. Using this covariance estimate, we constructed an inverse operator based on the sLORETA algorithm, incorporating a loose orientation constraint (loose = 1.0) and depth weighting (depth = 0.8) to mitigate the inherent bias toward superficial sources. The inverse solution was computed assuming a signal-to-noise ratio of 3.0, corresponding to a regularization parameter of λ^2^ = 0.111. This procedure yields source time courses representing estimates of current density across the cortical volume. (For an example of sLORETA activity, see Figure 3).

#### 2.2.2 Cortical parcellation and network mapping

We reduced the source-localized EEG data to parcel-level time series to support network-level analyses. After computing the distributed inverse solution, we obtained voxel-wise source time courses in the template source space. We registered a combined version of the HCP MMP 2.0 (Glasser et al., 2016) for this space, which yielded 46 bilateral cortical parcels (see Supplementary Table 1). For each parcel, we identified all voxels assigned by the atlas-to-source mapping and extracted their orientation-resolved source time series, computed using the loose orientation constraint and depth weighting detailed earlier. We derived regional time courses by averaging the voxel-level time series within each parcel using an unweighted arithmetic mean, producing one representative signal per parcel. This approach preserves parcel-level fluctuations while reducing voxel-specific noise. The resulting 46 parcel-wise time series were the basis for all our subsequent analyses.

### 2.3 Biologically-informed neural mass model

**Figure 4:**
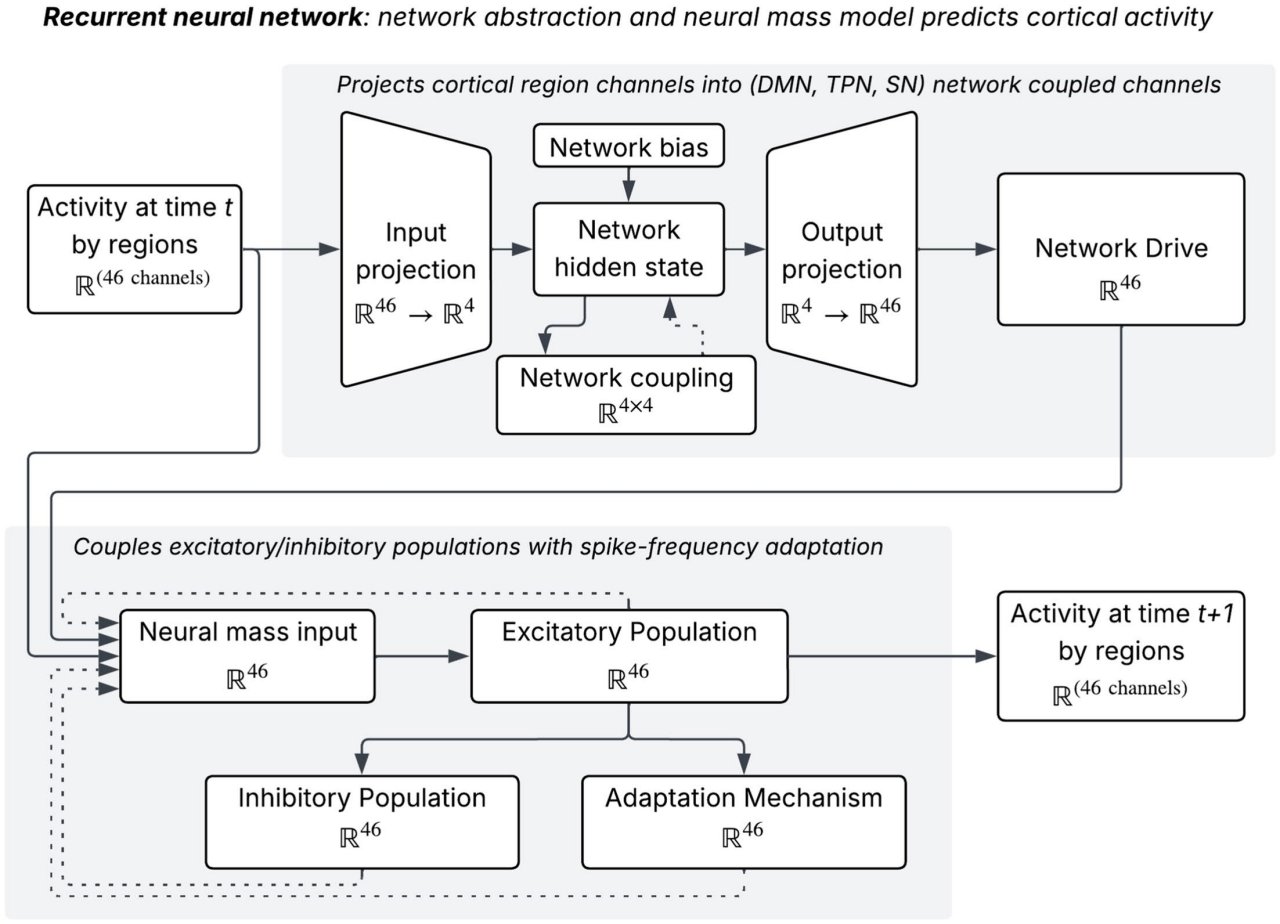
The recurrent network architecture consists of two components (network abstraction and neural mass). Network abstraction collects brain atlas regions into labeled functional networks using a mask. A latent population of activations by network is used as input into the neural mass which consists of excitatory, inhibitory, and adaptive populations.

To infer directional effective connectivity, we introduced the Biologically-Informed RNN (BIRNN) for source-localized neural mass modeling. The BIRNN is a predictive and interpretable recurrent framework that embeds neurobiologically grounded constraints directly into its architecture. It combines neural-mass dynamics at the source level with recurrent interactions to model directed influences among cortical regions. The full model architecture is shown in Figure 4.

#### 2.3.1 BIRNN neural mass architecture and dynamics

We implemented neural-mass dynamics at the level of 46 source-localized cortical parcels (See Figure 4). The model receives the observed regional time series $x_{t}\in {\mathbb{R}}^{46}$ at time step t, where $x_{t}$ represents the source-level activity extracted from the distributed inverse solution. Across all time points, the data forms a matrix $X\in {\mathbb{R}}^{N\times 46}$, with N denoting the number of samples. For each cortical region, the model maintains four biologically grounded hidden states:

1. an excitatory population state $h_{E}\in {\mathbb{R}}^{46}$;
2. an inhibitory population state $h_{I}\in {\mathbb{R}}^{46}$;
3. a slow adaptation state $h_{A}\in {\mathbb{R}}^{46}$, which captures activity-dependent fatigue or after-hyperpolarization processes; and
4. large-scale network state $h_{\mathrm{net}}\in {\mathbb{R}}^{4}$, representing in this study the four macro-scale cortical networks that exert top-down influence on parcel dynamics.

The BIRNN implements a discretized system of coupled differential equations that approximate a neural-mass model. At each time step, the excitatory population of each parcel receives five sources of input: (i) the external observed activity $x_{t}$, (ii) top-down drive from the network states, (iii) recurrent excitation from other parcels, (iv) recurrent inhibition from inhibitory populations, and (v) subtractive adaptation.

$$I_{E}(t)=x_{t}+W_{\text{out}}h_{\mathrm{net}}(t)+W_{\mathrm{EE}}h_{E}(t)+W_{\mathrm{IE}}h_{I}(t)-\beta h_{A}(t)$$

Where $W_{\text{out}}\in {\mathbb{R}}^{46\times 4}$ linearly maps network activity to parcels assuming similar contributions from all parcels in a network (See Supplementary Table 1). $W_{\mathrm{EE}},W_{\mathrm{IE}}\in {\mathbb{R}}^{46\times 46}$ respectively encode excitatory-to-excitatory and inhibitory-to-excitatory coupling, and β∈ℝ is the adaptation parameter. These matrices constitute the directional *effective connectivity* that the model learns.

The excitatory and inhibitory populations evolve according to first-order neural-mass equations with distinct time constants:


$$\frac{dh_{E}}{\mathrm{dt}}=\frac{1}{\tau_{E}}(-h_{E}+\sigma (I_{E}(t)))$$

$$\frac{dh_{I}}{\mathrm{dt}}=\frac{1}{\tau_{I}}(-h_{I}+\sigma (W_{\mathrm{EI}}h_{E}(t)))$$

where σ is the sigmoid activation function that converts synaptic input into a firing rate and $W_{\mathrm{EI}}\in {\mathbb{R}}^{46\times 46}$ encodes excitatory-to-inhibitory coupling. The time constants $\tau_{E},\tau_{I}\in \mathbb{R}$ control how rapidly the populations respond, with inhibitory populations typically evolving more quickly.

The adaptation state evolves more slowly and provides negative feedback to the excitatory population:


$$\frac{dh_{A}}{\mathrm{dt}}=\frac{1}{\tau_{A}}(-h_{A}+h_{E})$$

where $\tau_{A}\in \mathbb{R}$ governs the timescale of adaptation. This mechanism stabilizes the excitatory dynamics and prevents runaway activity by introducing a slow fatigue-like process.

The excitatory population activity $h_{E}(t)$ represents the model’s one-step prediction of the observed regional signal. Because this excitatory state integrates both local recurrent drive and long-range inputs, learning the connectivity matrices $W_{\mathrm{EE}},W_{\mathrm{IE}},W_{\mathrm{EI}}$ allows the model to infer directional, time-resolved effective connectivity among cortical parcels. In this way, the BIRNN provides a biologically interpretable recurrent architecture that directly links source-localized EEG dynamics to a structured neural-mass model.

#### 2.3.2 Network-level abstraction

We now describe how the regional dynamics captured by $h_{E}$ are aggregated into a low-dimensional representation that captures interactions among canonical large-scale networks. This step provides a critical bridge between regional neural-mass dynamics and interpretable, systems-level effective connectivity. To summarize whole-brain dynamics at a level more amenable to neuroscientific interpretation, we have introduced a network state vector $h_{\mathrm{net}}\in {\mathbb{R}}^{4}$, corresponding to four canonical functional systems. At each time step, the regional excitatory activity $x_{t}$ is projected into this network space and combined with the previous network state through a recurrent update:


$$h_{\mathrm{net}}(t)=\tanh(Wx_{t}+W_{\mathrm{net}}h_{\mathrm{net}}(t-1)+b_{\mathrm{net}})$$

Here $W_{\text{in}}\in {\mathbb{R}}^{4\times 46}$ is a fixed projection matrix encoding the assignment of cortical parcels to the four canonical networks (see data in Supplementary Table 1) and $b_{\mathrm{net}}$ is a learnable bias. This projection ensures that the network variables represent coherent, anatomically grounded population signals rather than arbitrary linear combinations of parcels. The matrix $W_{\mathrm{net}}\in {\mathbb{R}}^{4\times 4}$ is the central interpretable parameter of the model. Each (i,j) entry of this matrix quantifies the directional influence of network j to network i, inferred from the time-resolved dynamics of the underlying neural-mass populations.

Since the updates are recurrent and constrained by neural mass dynamics, $W_{\mathrm{net}}$ describes model-based effective connectivity, i.e., dynamic, directed temporal dependencies learned to optimize prediction of network activity under biological constraints. Unlike explicit causal inference frameworks such as DCM (Friston et al., 2003), which test specific mechanistic hypotheses, our approach discovers connectivity patterns that best explain observed temporal evolution. Thus, the network-level abstraction distills hundreds of electrophysiological time courses into a small set of interpretable dynamical couplings. The network identities and parcel-to-network assignments are defined *a priori* based on the HCP MMP 2.0 parcellation and established functional-anatomical correspondences (Glasser et al., 2016). The input projection matrix $W_{\text{in}}$ is therefore fixed and not learned, reflecting the constraint that each parcel contributes primarily to its assigned canonical network. In contrast, the network-to-network coupling matrix $W_{\mathrm{net}}$ is learned from data and represents the directional effective connectivity that the model infers without predefined interaction patterns. This design choice balances anatomical realism with flexibility in discovering state-dependent network dynamics.

#### 2.3.3 Embedded neurobiological constraints

A key advantage of the BIRNN model is that its learned connectivity is shaped by mechanistic and anatomical constraints known from systems neuroscience. These constraints prevent the RNN from exploiting degenerate statistical solutions and ensure that its inferences are physiologically interpretable. The constraints are as follows:

1. *Dale’s Law*: All synaptic populations obey biologically correct sign constraints:

- Excitatory projections $W_{\mathrm{EE}},W_{\mathrm{EI}}$ are constrained to remain non-negative.
- Inhibitory-to-excitatory projections $W_{\mathrm{IE}}$ are constrained to remain non-positive.

This prevents the model from learning sign-inconsistent interactions that would be biologically implausible and would undermine the interpretability of the population dynamics.

2 *Biophysically grounded time constants:* The time constants $\tau_{E},\tau_{I},\tau_{A}$ are initialized using values from empirical and modeling literature (e.g., $\tau_{I}>\tau_{E}$, reflecting slower inhibitory kinetics). During learning, these parameters are restricted to lie within physiologically credible ranges corresponding to membrane and synaptic time scales. This ensures that the model’s recurrent dynamics reflect the characteristic temporal response profiles of excitatory, inhibitory, and adaptation processes.
3 *Anatomical priors and structural regularization:* To reflect the sparsity and locality that characterize human cortical connectivity, we apply a regularization term that biases both regional and network-level connectivity toward sparse patterns and penalizes implausible long-range couplings. This constraint reflects principles of structural connectomics, including wiring-cost minimization and spatial embedding.

Collectively, these constraints anchor the model in established neurobiology, allowing it to learn condition- and subject-specific deviations while preventing physiologically implausible solutions.

#### 2.3.4 Model training and connectivity inference

The BIRNN is trained in a next-step prediction framework: given the state at time t, the model predicts the excitatory population activity $h_{E}(t+1)$. The loss function is the mean squared error (MSE), computed as the average squared difference between the true excitatory activity $h_{E}$ and the model’s predicted activity h^E, combined with biological regularization:

L=MSE(hE(t+1),h^E(t+1))+λRbio

where $R_{\mathrm{bio}}$encodes Dale’s law penalties, time-constant constraints, and sparsity-promoting anatomical priors. Training is performed with the AdamW optimizer (learning rate $5\times 10^{-4}$) with a ReduceLROnPlateau schedule for 150 epochs.

**Figure 5:**
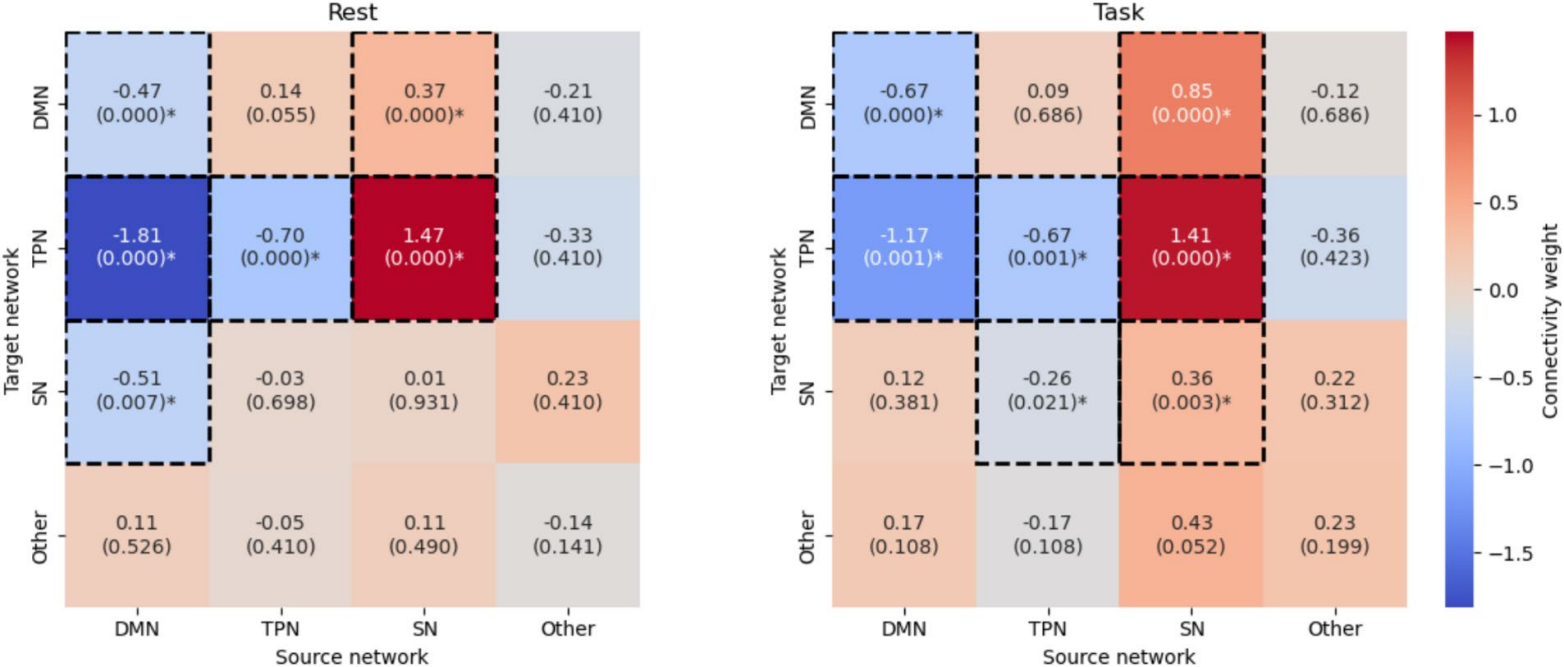
Mean network connectivity weights for task and resting conditions and corresponding statistical significance. Statistical significance is calculated as −log₁₀(FDR-corrected *p*-values) from one-sample *t*-tests (*N* = 15 subjects), with warmer colors indicating higher statistical significance. Values above the dashed line (corresponding to *q* < 0.05 after FDR correction) indicate connections that are reliably different from zero. The most statistically robust connections (*p* < 0.001 after FDR correction) were the facilitatory influences from the SN to both the TPN (SN → TPN: task mean = 1.41, *p*__FDR_ = 3.4 × 10^−6^; rest mean = 1.47, *p*_FDR_ = 6.1 × 10^−6^) and the DMN (SN → DMN: task mean = 0.85, *p*_FDR_ = 4.2 × 10^−5^; rest mean = 0.37, *p*_FDR_ = 3.3 × 10^−4^). The strong inhibitory coupling from TPN to DMN was also highly significant during rest (mean = −1.81, *p*_FDR_ = 6.1 × 10^−6^) and during task (mean = −1.17, *p*_FDR_ = 1.1 × 10^−3^). Self-inhibitory connections within DMN (task: *p*_FDR_ = 3.5 × 10^−4^; rest: *p*_FDR_ = 3.3 × 10^−4^) and TPN (task: *p*_FDR_ = 5.4 × 10^−4^; rest: *p*_FDR_ = 3.3 × 10^−4^) were also consistently significant across both states.

**Figure 6:**
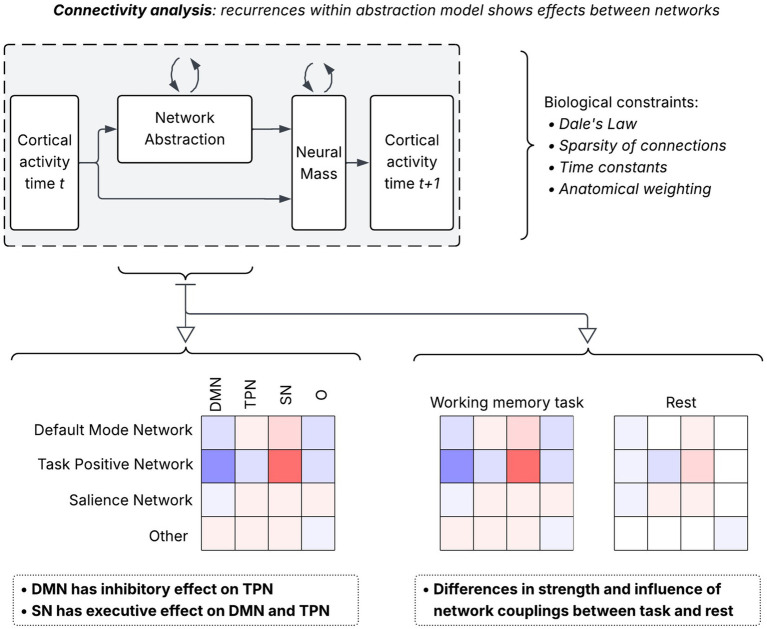
Connectivity analysis is done on the network abstraction component representing the functional network-level activities and covariances.

Although our model architecture is complex relative to the sample size (15 participants), we employed multiple complementary strategies to mitigate overfitting and ensure reliability of inferred connectivity patterns. First, the biological constraints embedded in our architecture substantially reduce the effective degrees of freedom: Dale’s law restricts connection signs, sparsity masks limit connectivity to 30% of possible connections, distance-dependent weighting biases local over long-range connections, and network membership constraints fix the parcel-to-network projection matrices *a priori*. These architectural constraints reduce the learnable parameter space by approximately 60% compared to an unconstrained recurrent network. Second, we implemented robust training regularization including early stopping with a patience of 20 epochs (restoring the best model state based on validation loss), adaptive learning rate scheduling (ReduceLROnPlateau with patience = 15, factor = 0.8), L1 regularization on connectivity matrices (*λ* = 0.001), and AdamW optimization with weight decay (1e-5). (see Results and Figure 5 for statistical reliability measures). Once the model has converged, the learned matrix $W_{\mathrm{net}}$ provides a compact and mechanistically interpretable summary of large-scale directional effective connectivity. Because each subject and condition is trained independently, the extracted $W_{\mathrm{net}}$ matrices reveal how the dynamic influence of one functional network on another is modulated by cognitive state or task engagement. These matrices constitute the primary output used in all downstream statistical and group-level analyses (see Figure 6).

#### 2.3.5 Statistical analysis of network connectivity

To assess the statistical reliability of the inferred network connectivity patterns, we performed one-sample t-tests for each of the 16 network-to-network connections (4 networks × 4 networks) in both the episodic memory task and resting state conditions. These tests evaluated whether the mean connectivity strength across participants (*N* = 15) was significantly different from zero. To control for multiple comparisons, we applied the Benjamini-Hochberg False Discovery Rate (FDR) correction (Benjamini and Hochberg, 1995) with a significance threshold of q < 0.05. The FDR procedure controls the expected proportion of false discoveries among rejected hypotheses, providing a less conservative but statistically rigorous approach than family-wise error rate corrections when testing multiple related hypotheses. With 16 comparisons per condition, this correction balances sensitivity for detecting true effects while maintaining control over Type I errors. The FDR-corrected *p*-values reported throughout represent the adjusted significance levels after accounting for the multiplicity of network connections tested.

#### 2.3.6 Baseline model validation

To validate that the biologically-constrained architecture provides explanatory advantages beyond interpretability, we compared the neural mass model against Granger Causality, an established baseline method for inferring directed dependencies. Granger Causality was applied to the same network-level time series (aggregated from 46 regions to 4 networks). We performed pairwise Granger causality analyses for all network pairs by fitting vector autoregressive models across lags 1–10. For each pair and each candidate lag, we tested whether including past values of the source time series significantly improved prediction of the target time series relative to a restricted model containing only the target’s own past. Statistical significance was assessed using F-tests on the joint contribution of the lagged source coefficients. For each pair, the lag corresponding to the minimum p-value across lags 1–10 was selected. This yielded F-statistics quantifying directed temporal dependencies. The baseline was applied to the same 15 participants and task/rest conditions as the neural mass model, enabling direct comparison.

#### 2.3.7 Association between connectivity patterns and imagery ability

We tested whether the inferred effective connectivity patterns relate to individual differences in visual imagery ability, thereby lending functional validity to our results. To that end, we regressed the weights reflecting most relevant connectivity identified by BIRNN onto VVIQ scores.

## 3 Results

### 3.1 BIRNN model results

#### 3.1.1 Behavioral data

Although our primary analyses focus on neural activity during the encoding phase, we briefly report behavioral recall performance to characterize task engagement. Participants averaged 11.27 (70%) correct recalls (SD = 2.91, range = 5–16) for word stimuli and 12.00 (75%) correct recalls (SD = 2.14, range = 8–16) for geometric shapes. VVIQ scores averaged 55.53 (SD = 11.18) and spanned the typical range (33–76) in the general population. Associations between recall rates and VVIQ accounted for < 2% (r = 0.14) of the variance.

#### 3.1.2 Connectivity patterns

We analyzed effective connectivity patterns inferred by the trained neural mass model across participants, comparing the memory task state (entire data) with the resting state. The resulting 4×4 effective connectivity matrices were aggregated across subjects using means. The neural mass model was trained separately for each participant on data from two primary conditions: the entire memory recall task and the resting state. Table 1 presents mean and median connectivity strengths for the aggregated connectivity matrices. Statistical significance testing using one-sample t-tests with FDR correction (see Methods 3.2.5) revealed that 7 of 16 connections were significant (q < 0.05) during the memory task, and 6 of 16 connections during rest (Figure 5).

**Table 1:** Mean and median network connectivity strengths across conditions.

Connection	Task (mean)	Task (median)	Rest (mean)	Rest (median)
DMN → DMN	−0.673*	−0.487	−0.474*	−0.385
DMN → TPN	0.092*	0.276	0.135*	0.132
DMN → SN	0.115	0.279	−0.506*	−0.456
DMN → Other	0.173	0.207	0.108	0.343
TPN → DMN	−1.171	−1.338	−1.806	−2.097
TPN → TPN	−0.672*	−0.493	−0.698*	−0.663
TPN → SN	−0.262*	−0.202	−0.033	−0.091
TPN → Other	−0.175	−0.216	−0.054	−0.056
SN → DMN	0.846*	0.734	0.369*	0.294
SN → TPN	1.409*	1.237	1.468*	1.363
SN → SN	0.362*	0.318	0.009	−0.092
SN → Other	0.429	0.739	0.112	0.068
Other → DMN	−0.124	0.230	−0.209	0.170
Other → TPN	−0.361	0.425	−0.333	−0.320
Other → Salience	0.221	0.298	0.225	0.365
Other → Other	0.226	0.443	−0.144	−0.220

Note: Positive values indicate net excitatory influence, negative values indicate net inhibitory influence. Values represent effective connectivity strengths extracted from the biologically-informed neural mass model. Asterisks * indicate statistical significance (*p* < 0.05).

In addition, Figure 5 provides a visual representation of these network dynamics.

SN showed strong excitatory influence on both DMN and TPN during the task state. The SN → DMN coupling was notably stronger during task (mean = 0.846) compared to rest (mean = 0.369), while SN → TPN coupling remained consistently high across both states (task mean = 1.409, rest mean = 1.468).

On the other hand, the TPN → DMN inhibitory connection was consistently strong across both states, but surprisingly stronger during rest (mean = −1.806) than during task (mean = −1.171). This pattern suggests that TPN-mediated suppression of DMN is not solely a task-specific phenomenon.

Finally, the DMN showed a striking reversal in its influence on the SN between states. During task, DMN → SN coupling was weakly positive (0.115), but during rest it became strongly negative (−0.506), suggesting state-dependent modulatory relationships.

Analysis of the individual participant data showed only a minor influence of outliers, and modest association between magnitude of the effects and individual variability. The observed state-dependent network reconfigurations showed considerable intraindividual consistency, confirming the reliability of the patterns.

Throughout training, Dale’s law constraints were preserved, with excitatory connections remaining positive and inhibitory connections negative. Time constants also remained within physiologically realistic ranges (excitatory: ∼5–30 ms, inhibitory: ∼10–100 ms), confirming that the learned dynamics reflected plausible neural mechanisms.

### 3.2 Baseline model validation

**Figure 7:**
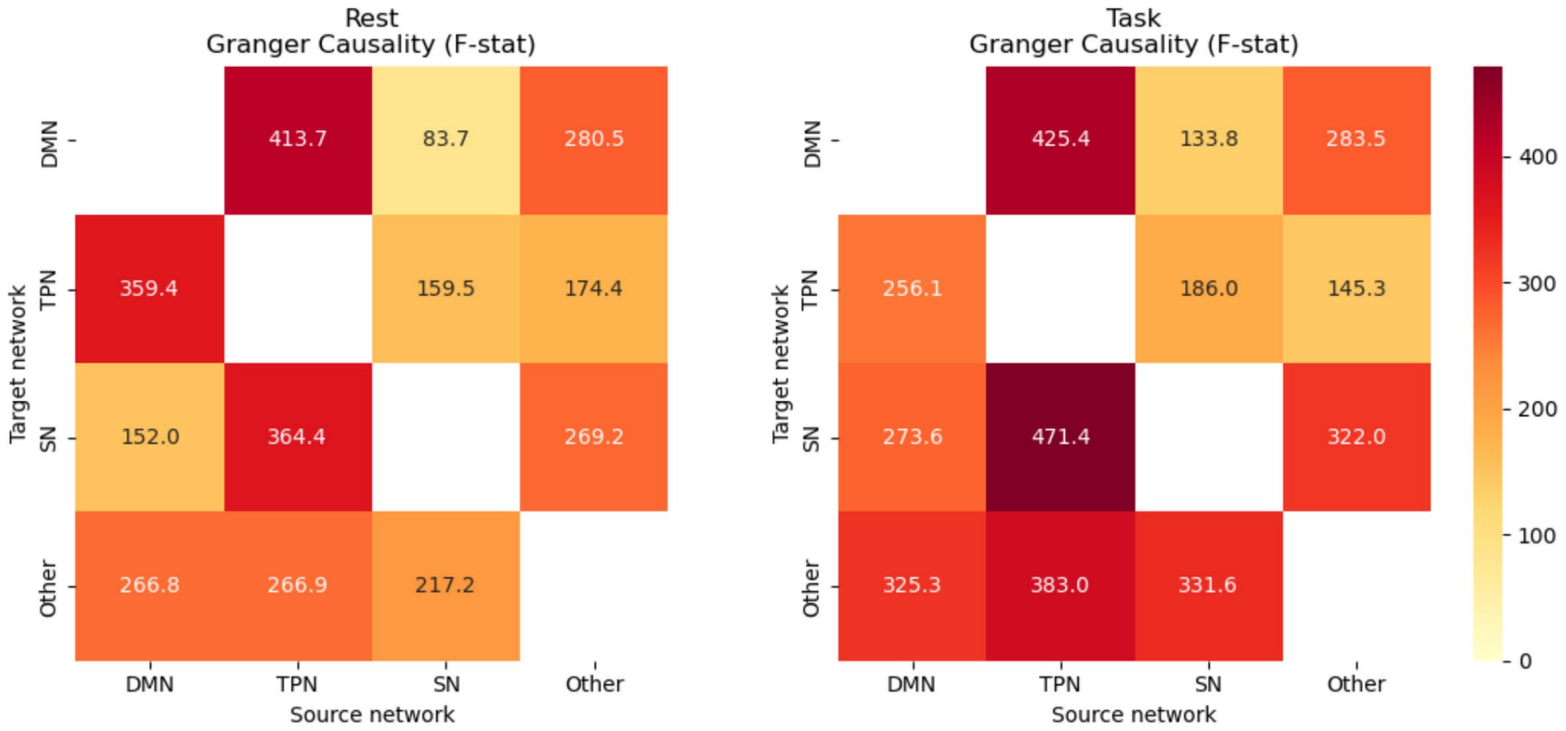
Granger causality for connectivity estimation. Granger causality F-statistics (log-scale) for rest (left) and task (right) show significant directed dependencies for major network pairs but provide no information about excitatory versus inhibitory mechanisms. Neural mass model connectivity matrices for the same conditions resolve signed, physiologically interpretable interactions, revealing that TPN → DMN is inhibitory (negative, consistent across states) while SN → TPN and SN → DMN are facilitatory (positive, stronger during task). Values shown are means across 15 participants.

To validate the necessity of biological constraints, we compared the neural mass model against Granger Causality (Figure 7). We performed pairwise Granger causality tests for all network pairs using grangercausalitytests function from statsmodels library, testing lags 1–10 and selecting the lag with the minimum *p*-value. For each ordered pair of networks (source j, target i), the test assessed whether the past activity of network j significantly improves prediction of network i beyond its own history. The resulting F-statistic was placed in position [i, j] of a 4 × 4 directed connectivity matrix, where each entry quantifies the strength of temporal dependence from source to target. Diagonal elements (self-connections) are undefined and set to zero by convention. This procedure was applied independently for each participant across both the task and resting-state conditions (*N* = 15), yielding subject-level F-statistic matrices that were subsequently averaged and tested for group-level significance using one-sample t-tests, enabling direct comparison with the neural mass model outputs.

Granger analysis detected statistically significant directed connectivity for all major network pairs (*p* < 0.05, see Supplementary Table 2), confirming that the observed patterns are not artifacts of model architecture. However, two critical limitations emerged: One underscores mechanistic ambiguity and the other highlights SN as “functional hub.

1. *Mechanistic Ambiguity*: Granger Causality provides only unsigned F-statistics, unable to distinguish excitatory facilitation from competitive inhibition. For example, while Granger detected strong TPN → DMN coupling (*F* = 425.4 during task, *F* = 413.7 at rest), it cannot resolve whether this represents competitive suppression (as the neural mass model reveals: mean = −1.17 during task) or cooperative facilitation.
2. *Salience Network Hub Function*: Granger Causality showed strong statistical dependencies for SN → TPN (*F* = 471.4, *p* < 0.001) and SN → DMN (*F* = 133.8, *p* < 0.05), but cannot determine the sign of these influences. The neural mass model reveals these are strong *positive* facilitatory connections (SN → TPN: +1.41, SN → DMN: +0.85), identifying the Salience Network as a modulatory hub rather than a competitive suppressor.

These results demonstrate that while Granger Causality validates that directed connectivity exists, only the biologically-constrained neural mass model captures physiologically interpretable network interactions detailing explicit excitatory/inhibitory mechanisms.

### 3.3 Correlation between connectivity and imagery

Given that BIRNN identified the SN → TPN coupling as the most central, we conducted an exploratory, hypothesis-generating correlational analysis to examine possible relationships with imagery ability, as measured by the VVIQ. Given the small sample size (N = 15) and post-hoc selection of connections, these analyses should be interpreted cautiously as preliminary observations requiring independent replication. We performed two separate robust non-linear regressions, first correlating VVIQ to the hidden connectivity strength weights for resting and the entire task, and then to the disaggregated weights for geometrical shape and word data. All regressions showed very modest correlations for rest, the entire task data and the geometric shape recall conditions (all R^2^ ≤ 0.09). However, for the word recall, VVIQ increased with the strength of SN → TPN (R^2^ = 0.25 (r (14) = 0.51); Simes-Bonferroni corrected *p* < 0.05). Another exploratory analysis on the DMN and TPN bidirectional connectivity showed a strong correlation (R^2^ = 0.41 (r (14) = 0.64); Simes-Bonferroni corrected *p* < 0.01) between VVIQ and DMN → TPN for the shapes recall, a relationship which appears to be mediated by SN.

## 4 Discussion

This study leveraged a biologically constrained neural-mass model to learn model-based effective connectivity from source-localized EEG, yielding a mechanistically interpretable account of large-scale network dynamics during episodic memory retrieval. By moving beyond correlational metrics to estimate directional causal interactions among the SN, TPN, and DMN, our findings challenge the traditional view of the triple network model as a set of discrete, switch-like modules. Instead, they strongly support the interpretation that the system operates as a continuously self-organizing, dynamically coupled architecture (Sridharan et al., 2008).

A key theoretical implication concerns the role of the SN. Rather than serving as a binary controller that allocates processing between DMN and TPN, the SN consistently exhibited facilitatory influences on both networks across cognitive states. The strong and stable SN → TPN couplings during memory retrieval (1.409) and rest (1.468), together with positive SN → DMN coupling during the task (0.846), identify the SN as a global modulatory hub that regulates gain across large-scale networks. These results are more consistent with self-organization accounts in which the SN maintains a metastable, readiness-enhancing network configuration, contradicting models that cast it as a competitive network switch.

The DMN–TPN relationship also reflected context-dependent emergent dynamics. Although TPN → DMN inhibition appeared in both states, its greater magnitude at rest (−1.806 vs. –1.171) suggests that this coupling reflects an intrinsic organizational baseline rather than a purely task-driven mechanism. The TPN inhibition of the DMN may therefore help structure the system’s attractor landscape, stabilizing transitions between internally and externally oriented cognition (D’Angiulli et al., 2024).

The most striking state-dependent reconfiguration emerged in the DMN → SN pathway, which shifted from weakly facilitatory during memory retrieval (0.115) to strongly inhibitory at rest (−0.506). This decisive polarity reversal indicates that the DMN does not simply disengage during rest; it actively down-regulates salience processing to stabilize an internally focused cognitive regime. This supports contemporary theories that view the resting state as a deliberately stabilized organizational mode rather than a passive baseline (Lefebvre and D’Angiulli, 2019).

Our baseline validation against Granger Causality confirmed that standard statistical approaches detect directed connectivity but cannot resolve critical organizational principles. Granger analysis successfully identified the same network pairs showing significant interactions, validating that our findings reflect genuine temporal dependencies rather than model artifacts. However, the unsigned F-statistics cannot distinguish excitatory facilitation from competitive inhibition—a fundamental limitation when interpreting network dynamics. Most critically, Granger Causality cannot reveal that the Salience Network exerts strong *positive* influences on both DMN and TPN, or that DMN → Salience undergoes a polarity reversal between states. These mechanistic insights emerge only when biological constraints (Dale’s Law, excitatory/inhibitory separation) are enforced, demonstrating that model complexity here purchases interpretability rather than merely fitting noise.

These findings demonstrate that biologically informed, generative modeling can reveal neural mechanisms completely invisible to correlation-based approaches. By enforcing Dale’s Law and physiologically realistic excitatory–inhibitory dynamics, the inferred connectivity reflects true neural operations. The results provide evidence that cognitive states emerge from self-organizing interactions across large-scale brain networks, with network-to-network coupling patterns inferred in a data-driven manner from anatomically-defined network structures (Pham et al., 2023).

Finally, preliminary exploratory analyses suggest the observed connectivity patterns may relate to individual differences in visual imagery ability, a psychological factor implicated in episodic encoding and retrieval. These exploratory findings, based on a modest sample size and post-hoc connection selection, should be considered hypothesis-generating rather than confirmatory. Participants with stronger imagery ability showed enhanced integration within the DMN and stronger DMN → TPN facilitation during the memory task, tentatively suggesting that imagery-based strategies may shape how attractor states are formed and stabilized during recall and its interaction with perception (D’Angiulli et al., 2024). This aligns with theoretical accounts proposing that vivid mental imagery relies on the coordinated recruitment of internally oriented (DMN) and control-related (TPN) processes, and may reflect a more flexible or metabolically efficient configuration of the underlying network architecture (D’Angiulli and Roy, 2024).

Taken together, the results provide preliminary support for a revision of the Triple-Network Model toward a more graded, bidirectional, self-organizing framework, wherein cognitive states—and individual differences in psychological abilities such as imagery—emerge from dynamic adjustments in inter-network coupling. The clear structure observed at rest further indicates that resting-state connectivity might reflect an active organizational regime, cautioning against interpreting it as a neutral baseline. More broadly, this work demonstrates how effective connectivity modeling can bridge neurobiological mechanisms, network-level organization, and meaningful psychological variability.

Several methodological considerations merit acknowledgment. The learned connectivity represents *predictive dependencies* optimized for temporal forecasting rather than causal mechanisms verified through perturbation of the system. Unlike DCM, our model does not distinguish between direct network influences and indirect effects mediated through unobserved pathways. The connectivity patterns reflect the overall temporal dependency structure constrained by neural mass dynamics. Despite these limitations, our approach offers valuable middle ground between black-box prediction and explicit hypothesis testing, revealing interpretable patterns of network coordination through biologically constrained learning. Hence, our approach makes it possible to give precise quantitative descriptions and predictions of dynamic connections that are much more complex and rich than sets of static one-way dependencies.

## Data Availability

The raw data supporting the conclusions of this article will be made available by the authors, without undue reservation.
