# Supplementary figures and images for: Neural self-organization during episodic encoding: deep recurrent effective connectivity from source-localized EEG
Source: Front Psychol. 2026 Mar 23;17:1766795. doi: 10.3389/fpsyg.2026.1766795 (PMC13050779; doi:10.3389/fpsyg.2026.1766795)

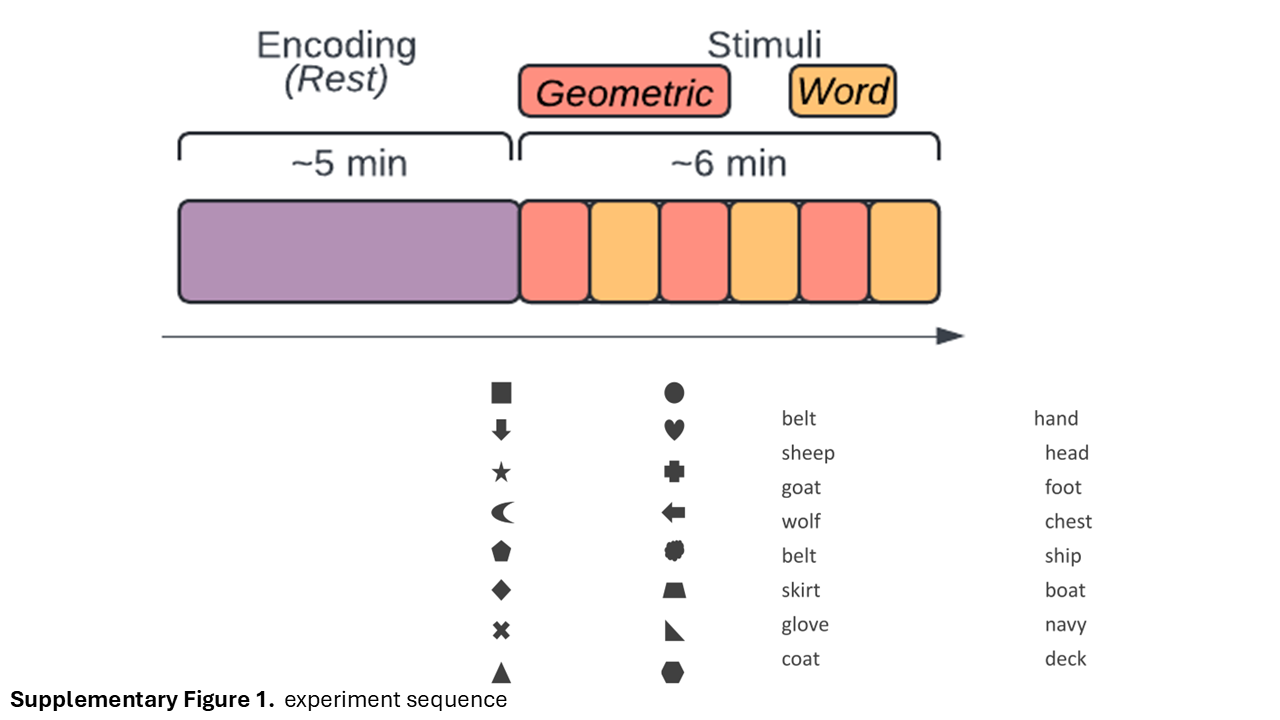

Supplement: Supplementary file 1 [file Image_1.TIF]

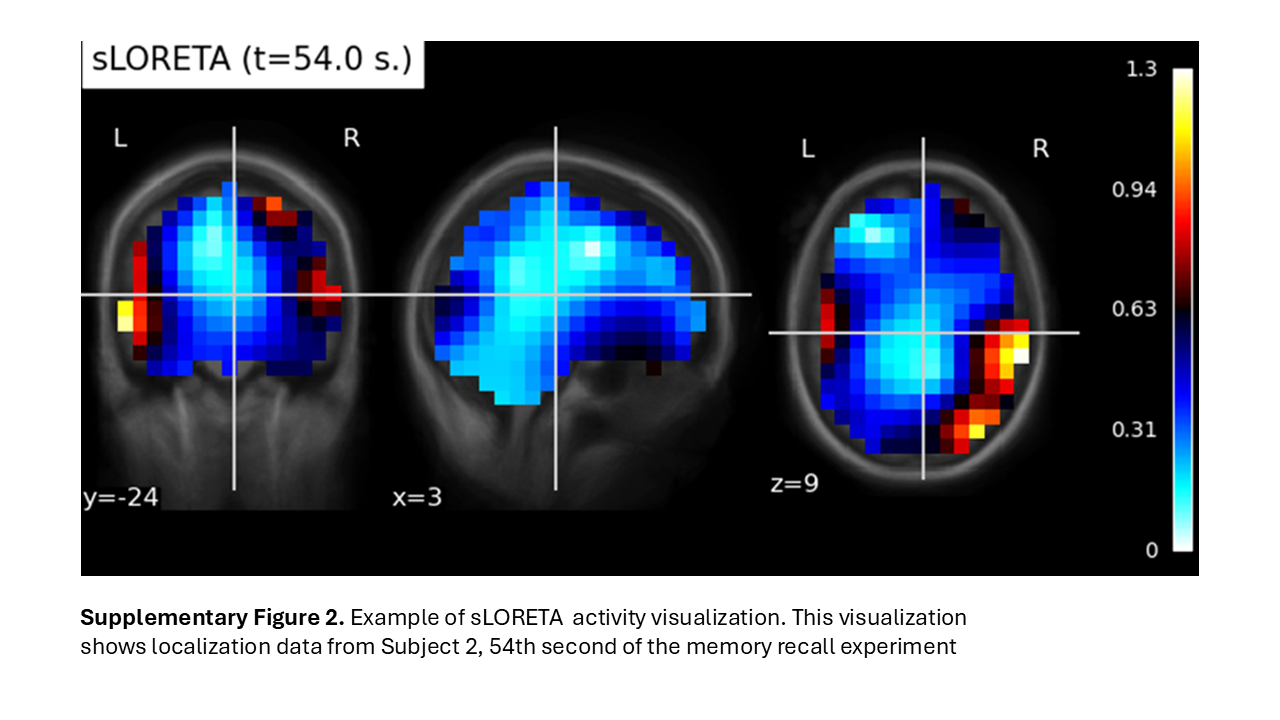

Supplement: Supplementary file 2 [file Image_2.TIF]
