# Supplementary material for: Neural self-organization during episodic encoding: deep recurrent effective connectivity from source-localized EEG
Source: Front Psychol. 2026 Mar 23;17:1766795. doi: 10.3389/fpsyg.2026.1766795 (PMC13050779; doi:10.3389/fpsyg.2026.1766795)
Supplement: Supplementary file 3 [file Table_1.DOCX]

***Supplementary Table 1.*** *Mapping of the 46 Cortical Regions to the Four Canonical Networks Used in the Model*

| Network | Associated Cortical Regions |
| --- | --- |
| DMN | Anterior Cingulate/Medial Prefrontal Cortex |
|  | Inferior Parietal Cortex |
|  | Lateral Temporal Cortex |
|  | Medial Temporal Cortex |
|  | Orbital and Polar Frontal Cortex |
|  | Posterior Cingulate Cortex |
|  | Temporo-Parieto-Occipital Junction |
| TPN | Auditory Association Cortex |
|  | Dorsal Stream Visual Cortex |
|  | DorsoLateral Prefrontal Cortex |
|  | Early Auditory Cortex |
|  | Early Visual Cortex |
|  | Inferior Frontal Cortex |
|  | MT+ Complex and Neighboring Visual Areas |
|  | Paracentral Lobular/Mid Cingulate Cortex |
|  | Posterior Opercular Cortex |
|  | Premotor Cortex |
|  | Primary Visual Cortex (V1) |
|  | Somatosensory and Motor Cortex |
|  | Superior Parietal Cortex |
|  | Ventral Stream Visual Cortex |
| SN | Insular and Frontal Opercular Cortex |
| Other | Unknown Region |

**Note.** Each listed region includes bilateral parcels (left and right hemispheres).
