# Supplementary material for: Neural self-organization during episodic encoding: deep recurrent effective connectivity from source-localized EEG
Source: Front Psychol. 2026 Mar 23;17:1766795. doi: 10.3389/fpsyg.2026.1766795 (PMC13050779; doi:10.3389/fpsyg.2026.1766795)
Supplement: Supplementary file 4 [file Table_2.docx]

**Supplementary Table 2. Granger Causality Validation Results**

Pairwise Granger causality F-statistics for all network connections during task (entire_data) and rest conditions. Values represent mean ± standard deviation across N=15 participants. Statistical significance was assessed using one-sample t-tests against zero (H₀: no Granger causality). Diagonal elements (self-connections) are undefined and set to zero by convention.

|  |  | **F-statistic (Mean ± SD)** | **p-value** | **Significant** | **F-statistic (Mean ± SD)** | **p-value** | **Significant** |
| --- | --- | --- | --- | --- | --- | --- | --- |
| **DMN** | **DMN** | 0.0 ± 0.0 | — | — | 0.0 ± 0.0 | — | — |
| **TPN** | **DMN** | 425.4 ± 533.0 | 0.0098 | ✓ | 413.7 ± 432.4 | 0.0030 | ✓ |
| **Salience** | **DMN** | 133.8 ± 189.4 | 0.0193 | ✓ | 83.7 ± 121.4 | 0.0219 | ✓ |
| **Other** | **DMN** | 283.5 ± 340.3 | 0.0076 | ✓ | 280.5 ± 385.0 | 0.0164 | ✓ |
| **DMN** | **TPN** | 256.1 ± 306.7 | 0.0075 | ✓ | 359.4 ± 536.3 | 0.0251 | ✓ |
| **TPN** | **TPN** | 0.0 ± 0.0 | — | — | 0.0 ± 0.0 | — | — |
| **Salience** | **TPN** | 186.0 ± 407.4 | 0.1096 | ✗ | 159.5 ± 275.8 | 0.0483 | ✓ |
| **Other** | **TPN** | 145.3 ± 201.7 | 0.0174 | ✓ | 174.4 ± 204.5 | 0.0065 | ✓ |
| **DMN** | **Salience** | 273.6 ± 359.7 | 0.0129 | ✓ | 152.0 ± 160.8 | 0.0033 | ✓ |
| **TPN** | **Salience** | 471.4 ± 366.4 | 0.0003 | ✓✓✓ | 364.4 ± 355.5 | 0.0018 | ✓✓ |
| **Salience** | **Salience** | 0.0 ± 0.0 | — | — | 0.0 ± 0.0 | — | — |
| **Other** | **Salience** | 322.0 ± 359.9 | 0.0048 | ✓ | 269.2 ± 407.6 | 0.0269 | ✓ |
| **DMN** | **Other** | 325.3 ± 422.7 | 0.0121 | ✓ | 266.8 ± 475.5 | 0.0544 | ✗ |
| **TPN** | **Other** | 383.0 ± 443.4 | 0.0060 | ✓ | 266.9 ± 310.4 | 0.0062 | ✓ |
| **Salience** | **Other** | 331.6 ± 556.9 | 0.0428 | ✓ | 217.2 ± 359.8 | 0.0404 | ✓ |
| **Other** | **Other** | 0.0 ± 0.0 | — | — | 0.0 ± 0.0 | — | — |

**Note**: Significance levels: ✓✓✓ denotes p < 0.001, ✓✓ denotes p < 0.01, ✓ denotes p < 0.05, ✗ not significant. F-statistics quantify the strength of Granger-causal influence from source to target network. Higher values indicate stronger temporal dependencies, but do not specify whether the influence is excitatory or inhibitory. DMN = Default Mode Network, TPN = Task-Positive Network.
